# Le nævus épidermique verruqueux inflammatoire linéaire

**DOI:** 10.11604/pamj.2015.22.177.7451

**Published:** 2015-10-22

**Authors:** Najwa Guerouaz, Badredine Hassam

**Affiliations:** 1 Service de Dermatologie, Vénérologie, Hôpital Ibn Sina, Rabat, Maroc

**Keywords:** Nævus épidermique, verrues inflammatoire, hyperplasies épidermiques, epidermal nevus, inflammatory warts, epidermal hyperplasia

## Image in medicine

Le nævus épidermique verruqueux inflammatoire linéaire (NEVIL) est une affection rare correspondant à des hyperplasies épidermiques bénignes qui se présentent cliniquement sous forme de lésions linéaires unilatérales hyperkératosiques. Une adolescente âgée de 16 ans, sans antécédents, présentait depuis la naissance une lésion, papuleuse brunâtre au niveau l'hémiface antérieure droite du cou avec une distribution Blaschko linéaire (a); et depuis 2 ans une lésion nodulaire kératosique verruqueuse, prurigineuse, grisâtre, d'aspect psoriasiforme par endroits au niveau de la région pariéto-occipitale droite du cuir chevelu (b). Le diagnostic de NEVIL était évoqué puis confirmé par la biopsie cutanée. Les dermocorticoïdes entraînaient une amélioration modeste. Les lésions cutanées résultent d'un phénomène de mosaïcisme génomique. Elles ont l'aspect de papules kératosiques verruqueuses, disposées en traînées, parfois reliées entre elles. Elles peuvent aussi se développer sur plusieurs territoires cutanés et sont parfois congénitale. L’évolution est chronique entrecoupée de poussées exsudatives et prurigineuses. Une extension secondaire des lésions est aussi possible comme chez notre patiente. Des anomalies diverses peuvent être associées comme l'aplasie d'une partie de membre. La distinction avec un psoriasis linéaire peut être difficile histologiquement. Les corticoïdes locaux sont peu efficaces, et l'exérèse chirurgicale est parfois pratiquée. Le laser CO2 trouve sa place dans cette indication avec des résultats satisfaisants.

**Figure 1:**
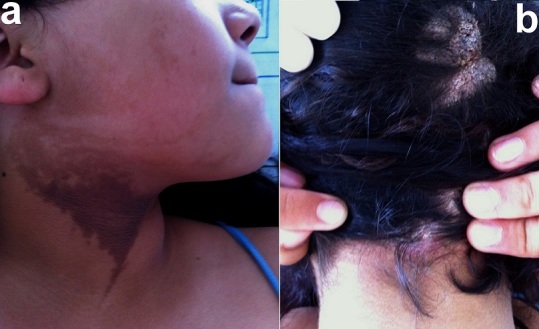
Nævus épidermique verruqueux inflammatoire linéaire du cou (a) et du cuir chevelu (b)

